# Assessment of Right Ventricular-Arterial Coupling by Echocardiography in Patients with Right Ventricular Pressure and Volume Overload

**DOI:** 10.31083/j.rcm2412366

**Published:** 2023-12-25

**Authors:** Hui Li, Teng Ye, Lan Su, Jue Wang, Zhijun Jia, Qilong Wu, Shusheng Liao

**Affiliations:** ^1^Department of Ultrasound, the First Affiliated Hospital of Wenzhou Medical University, 325000 Wenzhou, Zhejiang, China; ^2^Department of Cardiology, the First Affiliated Hospital of Wenzhou Medical University, 325000 Wenzhou, Zhejiang, China; ^3^Department of Cardiovascular Surgery, the First Affiliated Hospital of Wenzhou Medical University, 325000 Wenzhou, Zhejiang, China

**Keywords:** echocardiography, 3D, pulmonary hypertension, right ventricle-arterial coupling

## Abstract

**Background:**

Right ventricle-pulmonary arterial (RV-PA) coupling is considered the gold standard for assessing right ventricular (RV) function and can be evaluated noninvasively by echocardiography. The ratios of tricuspid annular plane systolic excursion/pulmonary artery systolic pressure (TAPSE/PASP), RV global longitudinal strain (G-RVLS)/PASP, and stroke volume/end-systolic volume (SV/ESV) have been proposed as surrogates of RV-PA coupling. The relationship of these parameters remains incompletely understood in patients with volume and pressure loading conditions. We aimed to compare these parameters and evaluate their relationship with 3D RV data in patients with RV pressure and volume overload.

**Methods:**

This study was performed on 110 individuals who underwent 2D and 3D echocardiography. Fifty-four patients had RV volume overload (atrial septal defect (ASD) group), 34 patients had RV pressure overload (pulmonary hypertension (PH) group), and 22 were controls. TAPSE/PASP, G-RVLS/PASP and SV/ESV ratios were calculated. Correlations between parameters of RV-PA coupling and 3D data were assessed using general linear mixed models.

**Results:**

Compared with the ASD group, the PH group had lower TAPSE/PASP and G-RVLS/PASP ratios. The SV/ESV ratio had a strong correlation with right ventricle ejection fraction (RVEF) in both ASD and PH patients (r = 0.8703, *p* < 0.001 and r = 0.9388, *p* < 0.001, respectively). The G-RVLS/PASP ratio showed a strong or moderately negative relationship with end-diastolic volume (EDV), ESV and SV (r = –0.7768, *p = *0.001; r = –0.7327, *p = *0.0005 and r = –0.6816, *p = *0.0018, respectively) in PH patients. The TAPSE/PASP ratio showed moderately negative correlations with EDV and ESV (r = –0.5712, *p = *0.0012 and r = –0.5594, *p = *0.0016, respectively) in PH patients.

**Conclusions:**

Non-invasive RV-PA coupling parameters derived from echocardiography appear similar, but not identical to profiles in pressure-overloaded and volume-overloaded patients. The correlations between non-invasive RV-PA coupling parameters and 3D data displayed various degrees of correlation.

## 1. Introduction

Pulmonary hypertension (PH) is a pathophysiological change resulting from various clinical entities [1]. Right ventricular (RV) morphology and function are very important determinants of the clinical presentation and prognosis in these diseases [2]. RV remodeling in response to different loading conditions includes volume or pressure overload [3]. Under physiological conditions, the pulmonary vascular bed maintains a relatively low resistance, and the RV contractility is matched to the pulmonary circulation, which is termed right ventricle-pulmonary arterial (RV-PA) coupling. RV-PA coupling can be invasively derived from the ratio between ventricular elastance and arterial elastance (Ees/Ea). Right heart catheterization (RHC) is the gold standard technique for evaluating RV-PA coupling, which directly acquires RV pressures and volumes [4, 5]. Nonetheless, costs and limited availability for the invasive nature of RHC may still limit the feasibility of RV function and morphology in patients on a daily basis.

Recently, several studies have indicated that RV-PA coupling can be evaluated noninvasively by echocardiography. The ratio of the tricuspid annular plane systolic excursion and pulmonary artery systolic pressure (tricuspid annular plane systolic excursion/pulmonary artery systolic pressure (TAPSE/PASP)) has been shown to have good correlation with invasive methods used to measure RV-PA coupling [4]. In addition, TAPSE/PASP was found to be a robust prognostic indicator in heart failure (HF) patients [6]. RV global longitudinal strain (G-RVLS)/PASP has also been employed to evaluate RV-PA coupling and was proven to have prognostic value in heart failure with reduced ejection fraction (HFrEF) patients [7].

Because of the complex geometry of the RV, two-dimensional echocardiography (2DE) parameters cannot fully reflect the overall function of the RV. Though cardiac magnetic resonance imaging (CMRI) is the gold standard image method for assessing RV volumes and function, it is limited by its expense and limited availability. With the progress in transducer technology, recent three-dimensional echocardiography (3DE) can provide a precise quantification of RV structure and function. Evaluation of the change in RV volume with 3DE may represent a novel approach for noninvasively evaluating RV-PA coupling. A previous study indicated that SV/ESV, as a volume estimate of RV-PA coupling, is an independent predictor of outcome in adult or pediatric PH patients [8, 9].

However, these three different methods of non-invasive RV-PA coupling parameters, TAPSE/PASP, RV global longitudinal strain (G-RVLS)/PASP and stroke volume/end-systolic volume (SV/ESV) ratio, have not been investigated in patients with different loading conditions. We aimed to compare the TAPSE/PASP, G-RVLS/PASP and SV/ESV ratios in patients with volume and pressure overload and to evaluate the relationship of these non-invasive RV-PA coupling ratios with RV functional and volumetric data derived from 3DE.

## 2. Methods

### 2.1 Study Patients

88 patients with chronic pressure or volume overload of the RV admitted to our hospital between December 2020 and November 2021 were enrolled in this study. 34 patients with chronic PH, defined by previous guidelines [10], formed the group with RV pressure overload. 54 patients diagnosed with a secundum atrial septal defect (ASD) constituted the chronic volume overload group. 22 healthy age-matched adults who had no history of cardiac or lung disease were selected as a control group. Patients with coronary artery disease, cardiomyopathy, significant arrhythmias (atrial fibrillation) or valvular heart disease (severe tricuspid regurgitation) were excluded.

This study was approved by local Institutional Ethics Committees in Clinical Research. All participants provided written informed consent for this research.

### 2.2 Echocardiographic Measurements and Analysis

In this study, standard transthoracic echocardiography examination was carried out using GE Vivid E95 and an M5S transducer (GE Healthcare, Norway). Digital images were stored for analysis offline. We measured routine parameters according to the ASE guidelines. Right heart linear parameters were measured on the RV-focused apical four-chamber view. RV fractional area change (FAC) was calculated as an index of RV function. TAPSE was acquired by M-mode echocardiography as another parameter of RV function. Systolic velocities (s’) of the tricuspid free wall annulus were measured by using Doppler tissue imaging. PASP was calculated as the sum of the tricuspid gradient and RAP, which was estimated using inferior vena cava diameter and collapsibility. For the Tei index of RV, we used the pulsed-wave tissue Doppler method to calculate the isovolumic time to the ejection time ratio on the lateral tricuspid annulus. We used pulse-wave spectral Doppler for estimating pulmonary acceleration time (PAT), which was defined as the interval between the onset of ejection and peak pulmonary flow velocity.

Longitudinal deformation of RV analysis was carried out offline using strain software, as described previously. Global longitudinal strain of the RV (G-RVLS) was calculated by averaging each segmental strain values of the RV free-wall and interventricular septum, and the RV free wall longitudinal strain (FW-RVLS) was equal to the average values of 3 regional strains.

**Fig. 1.**
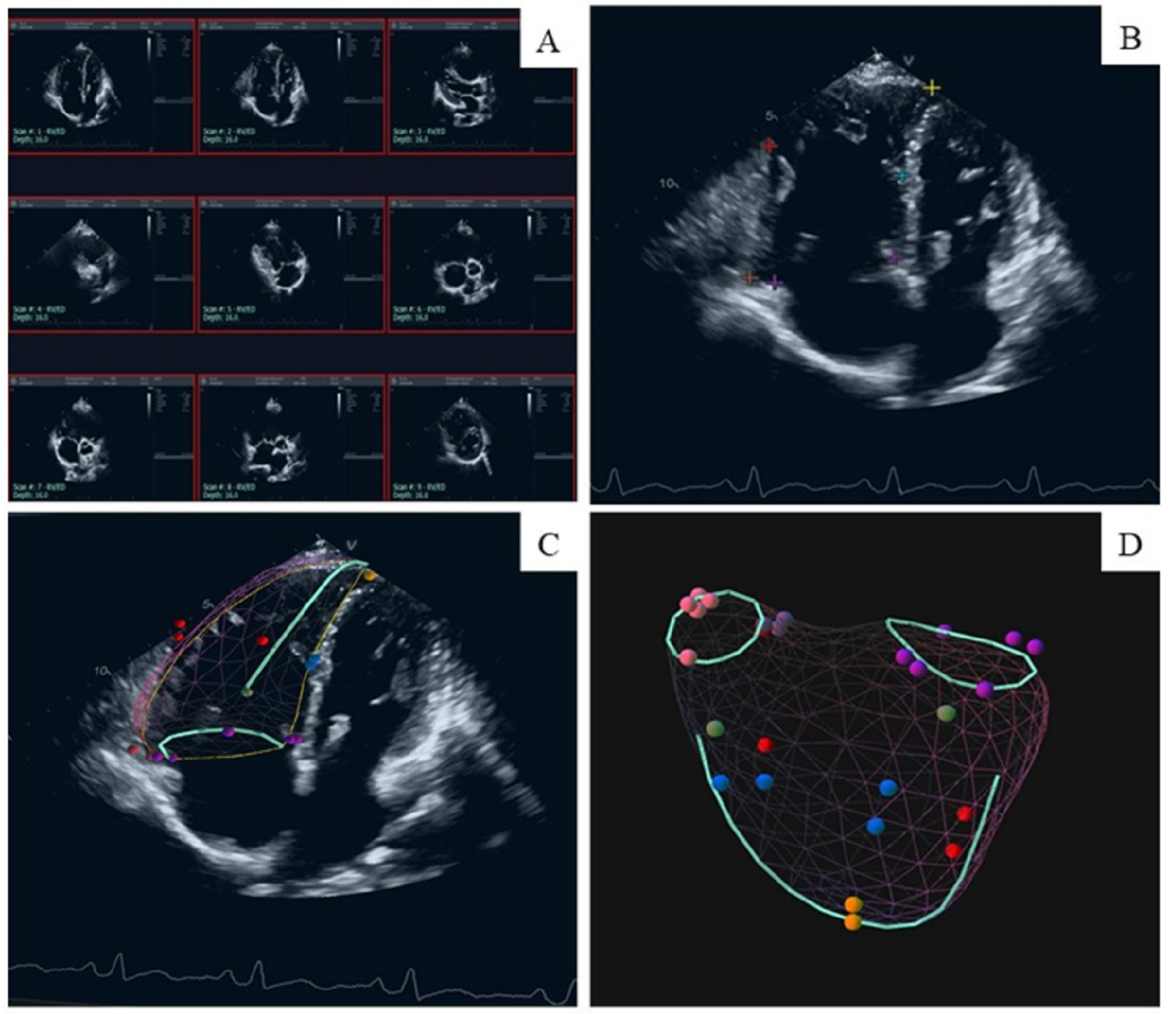
**Example of the stepwise process of right ventricular (RV) reconstruction by three-dimensional echocardiography (3DE)**. (A) Two-dimensional echocardiography B-mode plane of the right ventricle before entering 3DE mode. (B) Landmark setting in a preselected 2D image (Apical 4-chamber view) of the 3DE. (C) 3D model overlaid with 2D image (Apical 4-chamber view). (D) Model of an end-diastolic 3DE data set in a patient.

RV three-dimensional data were acquired from a new technique using a knowledge-based reconstruction (KBR) database, which has already been proven to correlate with the results in evaluating RV volumes from CMRI. We used a Ventrisound Analysis System (VAS) to perform the procedure as described previously [11]. The end-diastolic and end-systolic frames are automatically identified after the relevant two-dimensional echocardiographic image sections are transmitted to the system. Key anatomical locations of the right ventricle (at least 11 points are required) such as the tricuspid valve ring were marked on the two-dimensional section. After finishing the post-diastolic and post-systolic marking, the system automatically generated a three-dimensional model (Fig. 1A,B). For the accuracy of the results, the marked points can be deleted or added (Fig. 1C,D). Through the calculation of the 3D model, the relevant data of right heart function can be obtained, such as end-diastolic volume (EDV), end-systolic volume (ESV), stroke volume (SV), EF and cardiac output (CO). The body surface area indices of EDV, ESV, SV and CO were also measured.

TAPSE/PASP, G-RVLS/PASP, FW-RVLS/PASP and SV/ESV ratio were calculated as parameters of noninvasive RV-PA coupling.

### 2.3 Statistical Analysis

SPSS 24.0 for Windows (SPSS, Chicago, IL, USA) was used for statistical analysis. Continuous measurement data are expressed as mean ± standard deviation (SD). Student’s *t*-test was used to compare the counting data of ASD, PH and the control group. Pearson correlation coefficient was used to evaluate the correlation between RV-PA coupling parameters and RV 3D data.

20 objects were randomly selected for observer variation analysis of 3D data. The interval between readings required for internal variation testing was ≥14 days. Bland-Altman analysis was used to detect variability within and between observers.

## 3. Results

### 3.1 RV Size and Function in Patients and Controls

In all 54 ASD patients, the defect size ranged from 6 mm to 43 mm. 18 underwent device closure, and 36 received surgical repairs. The PH cohort included 21 patients with systemic lupus erythematosus (SLE), 5 with interstitial lung disease (ILD), 5 with chronic thromboembolic pulmonary hypertension (CTEPH) and 3 with idiopathic pulmonary arterial hypertension (IPAH).

Baseline demographics, clinical characteristics and right heart structure and function of the study cohort are summarized in Table 1. There were no statistically significant differences between patients and controls in age, BSA, SBP, and LVEF. The heart rate of ASD and PH patients was higher than that of controls. ASD and PH patients showed significantly larger right heart sizes than controls. Compared with the PH group, ASD patients demonstrated larger RV and RA diameters. RV FAC showed no significant differences between patients and controls. Compared with the control group, ASD and PH patients demonstrated significantly higher PASP, but there was no discrepancy between the ASD and PH groups. ASD patients showed significantly increased TAPSE compared with PH patients and controls. Similarly, ASD patients demonstrated significantly increased G-RVLS and FW-RVLS than PH patients and controls.

**Table 1.** **Baseline clinical characteristics, left and right heart size and function of the study patients and controls**.

Variable	ASD	PH	Controls	*p* value	*p* value	*p* value
	(N = 54)	(N = 34)	(N = 22)	ASD-PH	ASD-Controls	PH-Controls
Age (y)	42.38 ± 14.95	47.5 ± 13.91	43 ± 17.62	0.1073	0.8871	0.3184
Sex (female)	36 (66.7%)	27 (79.4%)	9 (37.5%)	0.0134	0.0001	0.0001
BSA ($m^{2}$)	1.63 ± 0.17	1.60 ± 0.16	1.67 ± 0.18	0.3669	0.4679	0.1799
HR (beats/min)	73.86 ± 10.30	75.53 ± 10.16	63.32 ± 10.53	0.4696	0.0003	0.0001
SBP (mmHg)	116.84 ± 12.35	116.37 ± 15.45	110.68 ± 12.74	0.8905	0.0631	0.1557
LVEF (%)	65.38 ± 3.99	64.53 ± 6.69	66.45 ± 3.33	0.5256	0.2362	0.1751
RA diameter (mm)	50.86 ± 5.87	41.75 ± 8.92	38.52 ± 4.46	<0.0001	<0.0001	0.1325
RA length (mm)	58.11 ± 10.62	50.17 ± 8.17	44.46 ± 4.64	0.001	<0.0001	0.0064
RA Area (${\mathrm{cm}}^{2}$)	24.81 ± 5.15	18.25 ± 6.50	14.46 ± 2.77	0.0002	<0.0001	0.0157
RV Basal diameter (mm)	48.74 ± 7.01	38.21 ± 7.25	35.28 ± 4.62	<0.0001	<0.0001	0.1129
RV middle diameter (mm)	42.89 ± 8.14	29.27 ± 7.75	27.79 ± 5.26	<0.0001	<0.0001	0.4570
RV long-axis diameter (mm)	78.51 ± 8.50	70.42 ± 7.71	64.94 ± 7.16	<0.0002	<0.0001	0.1741
FAC (%)	47.14 ± 4.98	43.30 ± 8.45	47.30 ± 5.63	<0.0569	0.9115	0.06874
PAT (ms)	111.8 ± 25.80	99.76 ± 37.36	145.1 ± 30.08	0.3684	0.1019	0.2491
TAV S’ (cm/s)	15.89 ± 4.19	16.53 ± 3.63	13.45 ± 3.81	0.4571	0.0188	0.0048
Tei index	0.35 ± 0.10	0.43 ± 0.12	0.41 ± 0.08	0.0152	0.0162	0.5634
PASP (mmHg)	46.67 ± 9.67	53.85 ± 17.59	27.4 ± 3.83	0.0343	<0.0001	<0.0001
TAPSE (mm)	27.94 ± 5.58	23.31 ± 5.52	22.95 ± 3.32	0.0004	<0.0001	0.7716
G-RVLS (%)	23.39 ± 3.99	18.21 ± 4.72	20.8 ± 2.83	0.0003	0.0046	0.0514
FW-RVLS (%)	27.07 ± 5.50	19.72 ± 5.88	24.57 ± 3.74	<0.0001	0.0399	0.0054

BSA, body surface area; HR, heart rate; SBP, systolic blood pressure; LVEF, left ventricular ejection fraction; FAC, fractional area change; PAT, pulmonary acceleration time; Tei index, RV myocardial performance index; TAV S’, tricuspid annular systolic velocity by tissue Doppler image; G-RVLS, RV global longitudinal strain; FW-RVLS, RV free wall longitudinal strain; ASD, atrial septal defect; PH, pulmonary hypertension; RA, right atrial; RV, right ventricular; TAPSE, tricuspid annular plane systolic excursion; PASP, pulmonary artery systolic pressure.

### 3.2 RV 3D Volumetric and Functional Indices in Patients and Controls

**Fig. 2.**
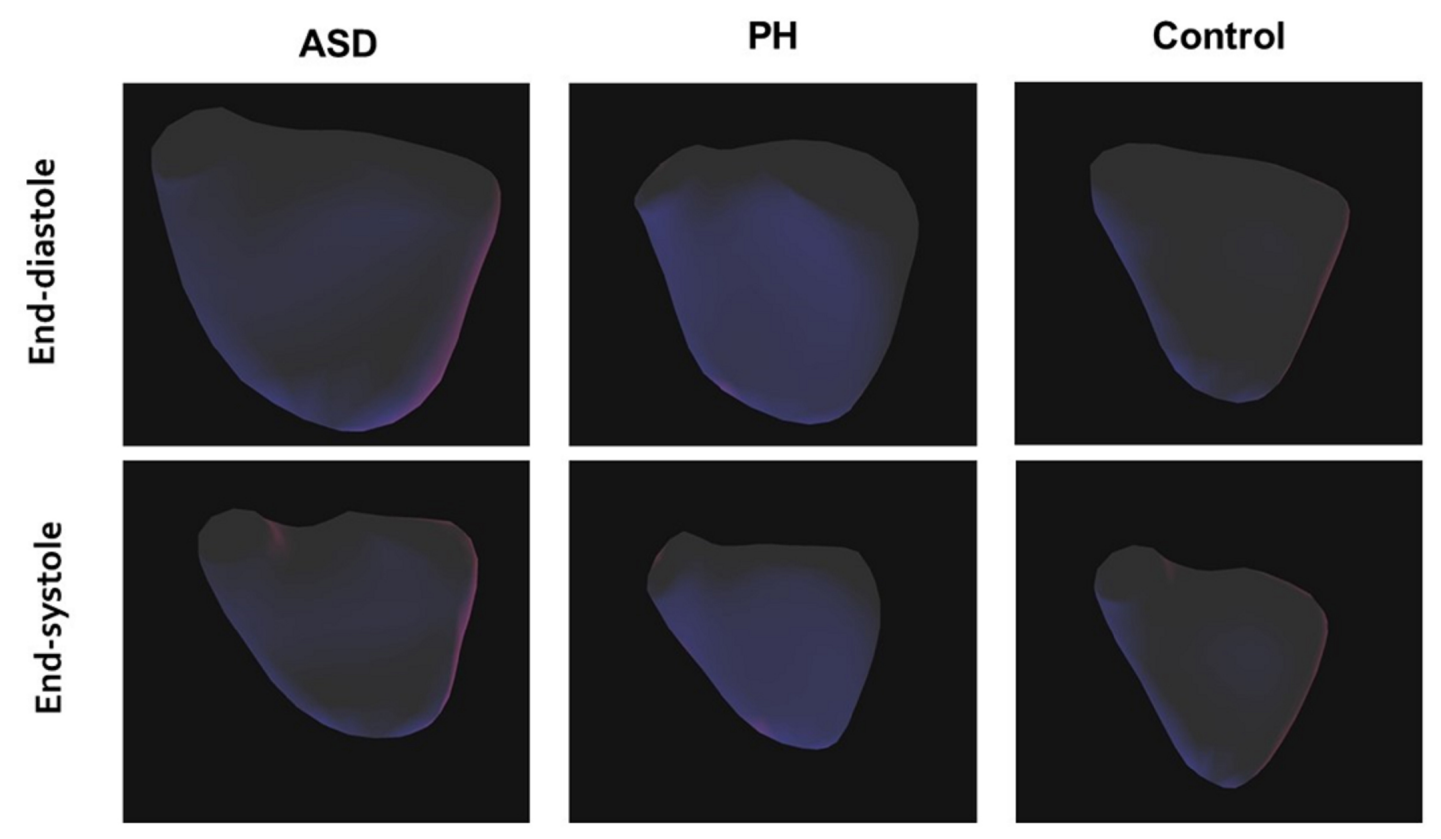
**Three-dimensional (3D) maps of the right ventricle in atrial septal defect (ASD) and pulmonary hypertension (PH) patients and healthy adults at end-diastole and end-systole**.

The RV three-dimensional parameters of the study cohort are presented in Table 2. Compared with the PH group, ASD patients showed significantly larger RV EDV (248.78 ± 87.98 vs 152.84 ± 56.87, *p* < 0.001), ESV (113.17 ± 45.73 vs 80.71 ± 40.58, *p* = 0.0015) and SV (138.99 ± 49.09 vs 75.87 ± 26.63, *p* < 0.0001), even after adjusting for BSA. Compared with the PH groups and controls, ASD patients showed significantly increased CO (10.01 ± 3.66 vs 5.53 ± 2.04 and 5.05 ± 1.42, *p* < 0.0001 and <0.0001, respectively) and CI (6.10 ± 2.08 vs 3.45 ± 1.22 and 3.00 ± 0.78, *p* < 0.0001 and <0.0001, respectively). However, PH patients demonstrated significantly lower RVEF than ASD patients and controls (49.76 ± 8.96 vs 56.31 ± 6.45 and 55.43 ± 6.54, *p* = 0.0011 and = 0.0128, respectively) (Fig. 2).

**Table 2.** **Comparison of 3D volumetric and functional indices between patients and controls**.

Variable	ASD	PH	Controls	*p* value	*p* value	*p* value
	(N = 54)	(N = 34)	(N = 22)	ASD-PH	ASD-Controls	PH-Controls
EDV (mL)	248.78 ± 87.98	152.84 ± 56.87	146.52 ± 36.05	<0.001	<0.0001	0.6374
ESV (mL)	113.17 ± 45.73	80.71 ± 40.58	65.72 ± 20.97	0.0015	<0.0001	0.0962
EDVi (mL/$m^{2}$)	147.94 ± 52.26	87.34 ± 27.27	87.00 ± 18.35	<0.001	<0.0001	0.9586
ESVi (mL/$m^{2}$)	66.69 ± 25.59	48.43 ± 20.68	38.81 ± 10.61	<0.0001	<0.0001	0.0376
SV (mL)	138.99 ± 49.09	75.87 ± 26.63	80.81 ± 19.64	<0.0001	<0.0001	0.4533
SVi (mL/$m^{2}$)	84.77 ± 28.69	47.01 ± 15.35	48.20 ± 11.21	<0.0001	<0.0001	0.7525
EF (%)	56.31 ± 6.45	49.76 ± 8.96	55.43 ± 6.54	0.0011	0.5999	0.0128
CO (L/min)	10.01 ± 3.66	5.53 ± 2.04	5.05 ± 1.42	<0.0001	<0.0001	0.3299
CI (L/min.$m^{2}$)	6.10 ± 2.08	3.45 ± 1.22	3.00 ± 0.78	<0.0001	<0.0001	0.1162

EDV, end-diastolic volume; ESV, end-systolic volume; EDVi, end-diastolic volume index; ESVi, end-systolic volume index; SV, Stroke volume; SVi, Stroke volume index; CO, Cardiac output; CI, Cardiac index; EF, ejection fraction; ASD, atrial septal defect; PH, pulmonary hypertension.

### 3.3 RV-PA Coupling Ratio by Echocardiography in Patients and Controls

**Fig. 3.**
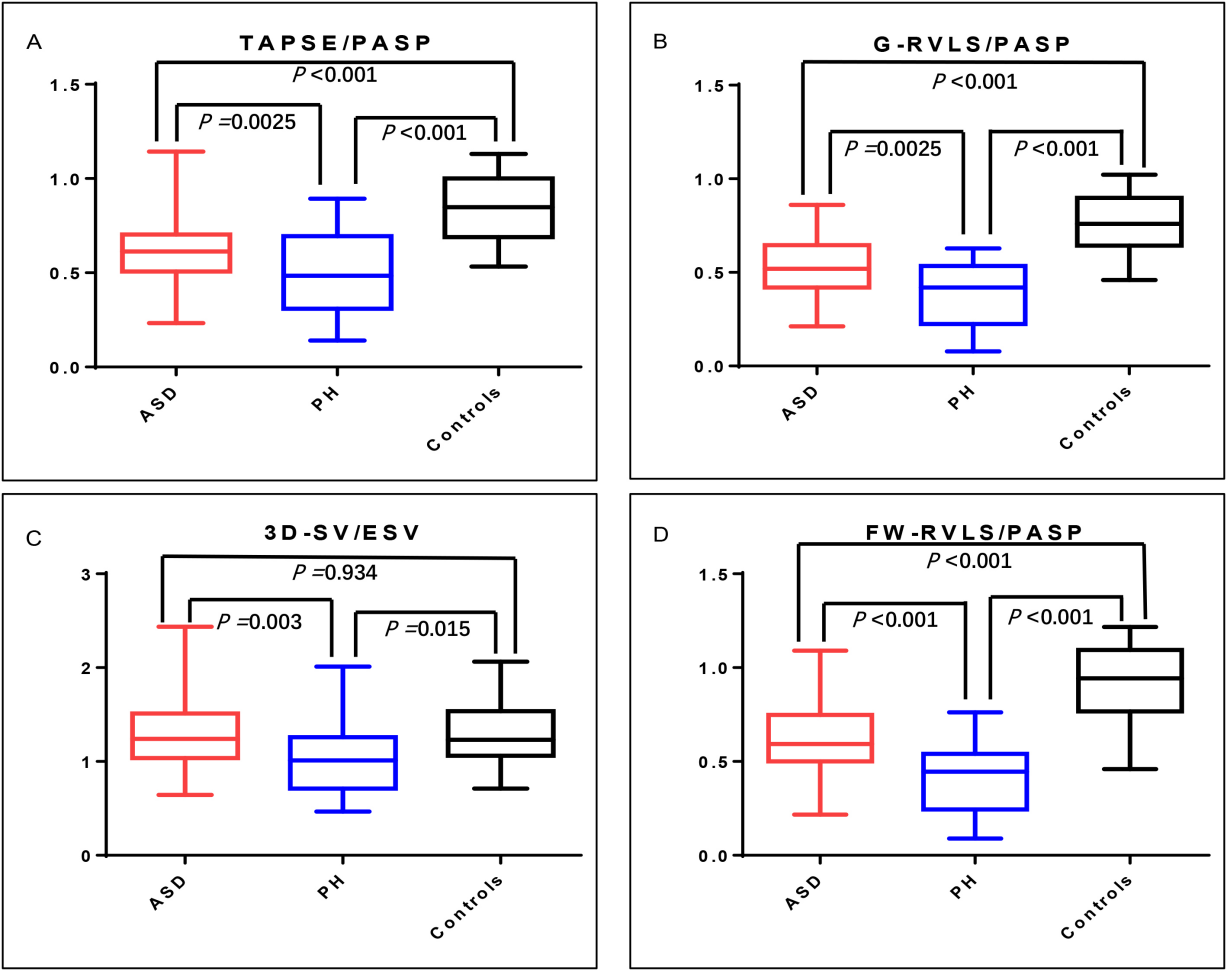
**Box-and-whisker plots of right ventricle-pulmonary arterial (RV-PA) coupling parameters in ASD patients, PH patients and controls**. A, B, C and D demonstrated the differences in RV-PA coupling parameters of TAPSE/PASP, G-RVLS/PASP, SV/ESV and FW-RVLS/PASP in patients and controls, respectively. The PH group showed the lowest scores in all three different modalities of RV-PA coupling parameters. ASD, atrial septal defect; PH, pulmonary hypertension. G-RVLS, right ventricle global longitudinal strain; FW-RVLS, right ventricle free wall longitudinal strain; SV, Stroke volume; ESV, end-systolic volume; 3D, three-dimensional; TAPSE, tricuspid annular plane systolic excursion; PASP, pulmonary artery systolic pressure.

Fig. 3 and Table 3 demonstrate the differences in RV-PA coupling parameters in patients and controls. ASD and PH patients had lower TAPSE/PASP, G-RVLS/PASP and FW-RVLS/PASP ratios than controls. Moreover, compared with the ASD group, the PH group had lower TAPSE/PASP, G-RVLS/PASP and FW-RVLS/PASP ratios. The PH group had a lower SV/ESV ratio than the ASD group and controls, but there was no statistically significant difference between the ASD group and controls.

**Table 3.** **Comparison of RV-PA coupling parameters in patients and controls**.

Variable	ASD	PH	Controls	*p* value	*p* value	*p* value
	(N = 54)	(N = 34)	(N = 22)	ASD-PH	ASD-Controls	PH-Controls
TAPSE/PASP	0.62 ± 0.17	0.49 ± 0.20	0.86 ± 0.18	0.0025	<0.0001	<0.0001
G-RVLS/PASP	0.53 ± 0.14	0.38 ± 0.17	0.79 ± 0.15	0.0025	0.0001	<0.0001
3D-SV/ESV	1.30 ± 0.36	1.03 ± 0.39	1.29 ± 0.34	0.0031	0.9342	0.0152
FW-RVLS/PASP	0.62 ± 0.18	0.41 ± 0.19	0.91 ± 0.20	0.0006	<0.0001	<0.0001

ASD, atrial septal defect; PH, pulmonary hypertension. G-RVLS, right ventricle global longitudinal strain; FW-RVLS, right ventricle free wall longitudinal strain; SV, Stroke volume; ESV, end-systolic volume; RV-PA, right ventricle-pulmonary arterial; 3D, three-dimensional; TAPSE, tricuspid annular plane systolic excursion; PASP, pulmonary artery systolic pressure.

### 3.4 RV-PA Coupling Ratio and 3D Parameter Relationship in Patients

**Fig. 4.**
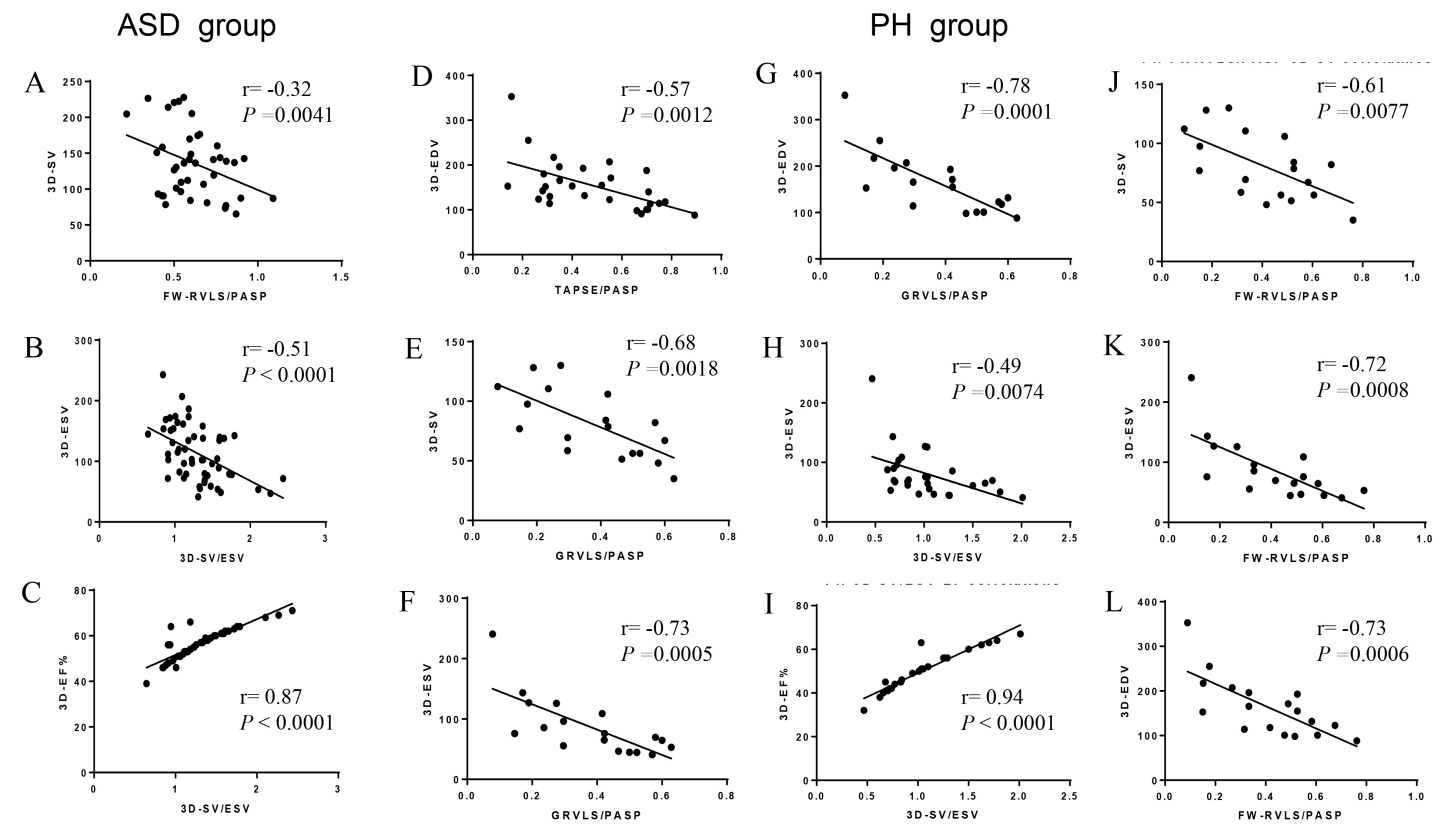
**Pearson correlation of right ventricle-pulmonary arterial (RV-PA) coupling parameters to RV three-dimensional (3D) data in the atrial septal defect (ASD) and pulmonary hypertension** (**PH) groups**. (A) The RV free wall longitudinal strain (FW-RVLS)/pulmonary artery systolic pressure (PASP) ratio to 3D-stroke volume (SV) demonstrated moderately negative correlations in ASD group. (B, D–H, J–L) The FW-RVLS/PASP, RV global longitudinal strain (G-RVLS)/PASP and 3D-SV/end-systolic volume (ESV) ratio to the relevant three-dimensional parameters showed a medium-high negative correlation in the ASD and PH groups. (C,I) The SV/ESV ratio in the ASD and PH groups was highly positively correlated with right ventricle ejection fraction (RVEF).

Table 4 and Fig. 4 demonstrated the relationships between non-invasive RV-PA coupling parameters and 3D data in ASD and PH patients. The SV/ESV ratio showed a strong correlation with RVEF in both ASD and PH patients (r = 0.8703, *p* < 0.001 and r = 0.9388, *p* < 0.001, respectively). The results showed moderate correlations between the SV/ESV ratio and ESV both in ASD and PH patients (r = –0.5073, *p* < 0.001 and r = –0.4871, *p* = 0.0074, respectively).

**Table 4.** **Correlations of RV-PA coupling parameters to 3D data in ASD and PH groups**.

Variable		3D-EF%		3D-EDV		3D-ESV		3D-SV	
		r	*p* value	r	*p* value	r	*p* value	r	*p* value
ASD Group	TAPSE/PASP	0.1953	0.1611	–0.1217	0.3853	–0.1548	0.2684	–0.04256	0.7622
	G-RVLS/PASP	–0.0934	0.5612	–0.2618	0.0982	–0.2554	0.107	–0.2868	0.0691
	3D-SV/ESV	**0.8703**	< **0.0001**	–0.1675	0.226	**–0.5073**	< **0.0001**	0.1161	0.4033
	FW-RVLS/PASP	–0.1038	0.5182	–0.2922	0.0638	–0.306	0.0517	**–0.3198**	**0.0041**
PH Group	TAPSE/PASP	0.2843	0.135	**–0.5712**	**0.0012**	**–0.5594**	**0.0016**	–0.3564	0.0577
	G-RVLS/PASP	0.2343	0.3493	**–0.7768**	**0.0001**	**–0.7327**	**0.0005**	**–0.6816**	**0.0018**
	3D-SV/ESV	**0.9388**	< **0.0001**	–0.1721	0.372	**–0.4871**	**0.0074**	0.3502	0.0626
	FW-RVLS/PASP	0.2892	0.2444	**–0.7258**	**0.0006**	**–0.7183**	**0.0008**	**–0.6063**	**0.0077**

ASD, atrial septal defect; PH, pulmonary hypertension; TAPSE, tricuspid annular plane systolic excursion; PASP, pulmonary artery systolic pressure; G-RVLS, right ventricle global longitudinal strain; FW-RVLS, right ventricle free wall longitudinal strain; SV, stroke volume; ESV, end-systolic volume; EDV, end-diastolic volume; RV-PA, right ventricle-pulmonary arterial; 3D, three-dimensional; EF, ejection fraction. The bold data are statistically significant.

The relationships between G-RVLS/PASP, FW-RVLS/PASP and 3D data were also analyzed. The G-RVLS/PASP ratio showed a negative relationship with EDV, ESV and SV (r = –0.7768, *p* = 0.0001; r = –0.7327, *p* = 0.0005 and r = –0.6816, *p* = 0.0018, respectively) in PH patients, but there was no correlation in ASD patients. Similarly, the FW-RVLS/PASP ratio also showed a negative relationship with EDV, ESV and SV (r = –0.7258, *p* = 0.0006; r = –0.7183, *p* = 0.0008 and r = –0.6063, *p* = 0.0077, respectively) in PH patients. But in ASD patients, it just showed a weak correlation with SV (r = –0.3198, *p* = 0.0041).

Regarding the TAPSE/PASP ratio, moderately negative correlations were found with EDV (r = –0.5712, *p* = 0.0012) and ESV (r = –0.5594, *p* = 0.0016) in PH patients. There was no correlation between RV 3D parameters and TAPSE/PASP ratio in ASD patients.

### 3.5 Reproducibility Results

**Fig. 5.**
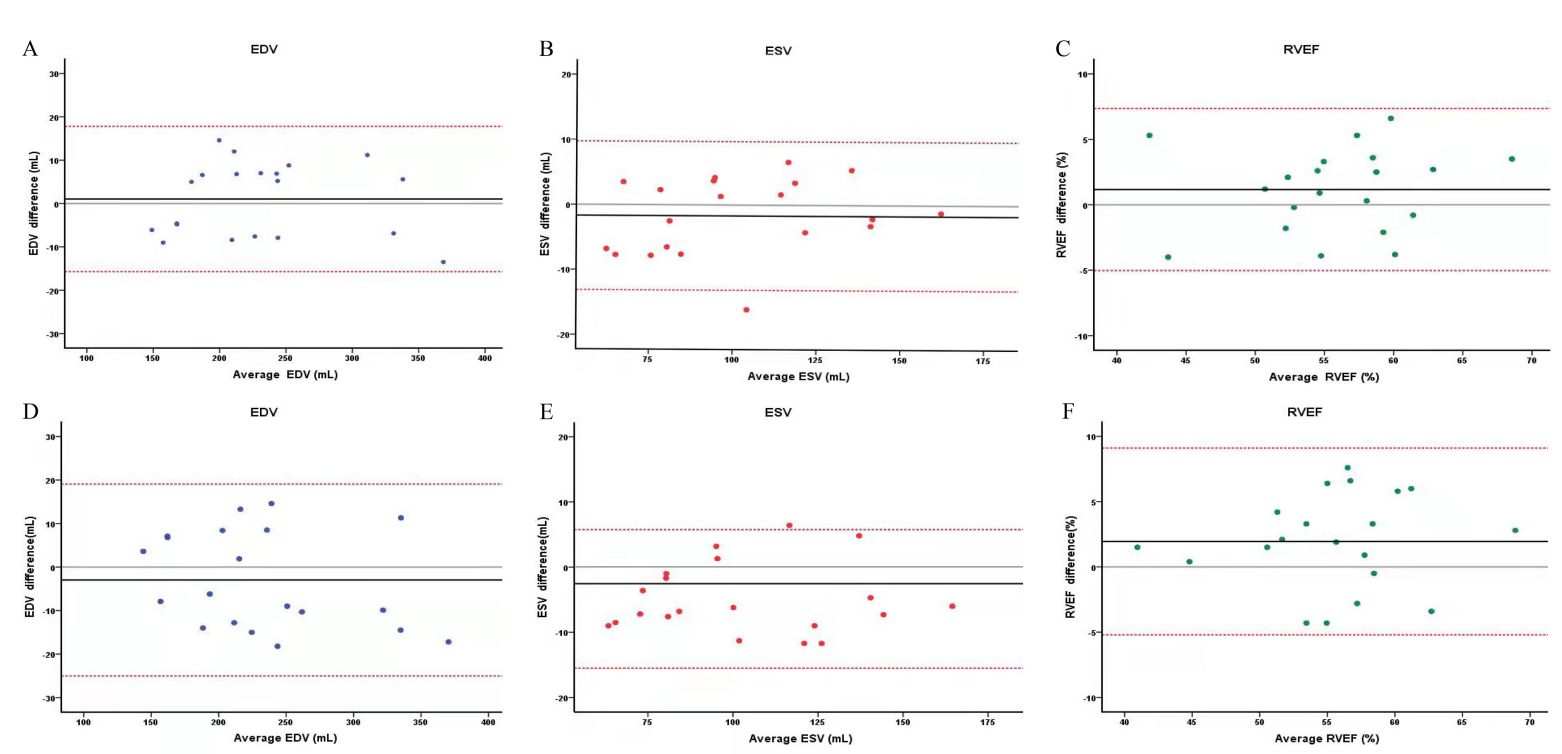
**Bland‒Altman analysis of intraobserver and interobserver variability for three-dimensional echocardiography (3DE) quantification of EDV, ESV, and RVEF in patients and control populations**. (A–C) Analysis bias of intraobserver variability in EDV, ESV, and RVEF, respectively. (D–F) Analysis bias of interobserver variability in EDV, ESV, and RVEF, respectively. EDV, end-diastolic volume; ESV, end-systolic volume; RVEF, right ventricle ejection fraction.

The intraobserver and interobserver variability results of EDV, ESV, and RVEF are shown in Fig. 5. The intraobserver variability for EDV was 1.05 ± 8.56 (95% CI, –15.73–17.82), and the interobserver variability was –2.98 ± 11.25 (95% CI, –25.03–19.08). The intraobserver variability for ESV was –1.69 ± 5.83 (95% CI, –13.12–9.75), and the interobserver variability was –4.88 ± 5.42 (95% CI, –15.50–5.74). The intraobserver variability for RVEF was 1.17 ± 3.16 (95% CI, –5.03–7.36), and the interobserver variability was 1.95 ± 3.65 (95% CI, –5.21–9.11).

## 4. Discussion

Our study demonstrated non-invasive RV-PA coupling parameters have profiles that are similar, but not identical in RV volume- and pressure-overloaded patients. The results show that (1) ASD patients had larger RV 3D volumetric indices and higher RVEF than PH patients. (2) Non-invasive RV-PA coupling parameters, such as TAPSE/PASP, G-RVLS/PASP and FW-RVLS/PASP ratios, decreased both in ASD and PH patients. Moreover, these ratios decreased more significantly in pressure-overloaded conditions (PH group). (3) The correlations between non-invasive RV-PA coupling parameters and 3D data displayed various degrees of correlation. The SV/ESV ratio derived from the 3D volumetric method showed a strong correlation with RVEF, as did the G-RVLS/PASP, FW-RVLS/PASP. However, the TAPSE/PASP ratio had only a moderate correlation with 3D parameters.

RV responds differently to pressure- and volume-overload conditions. There is remodelling in both conditions but relative preservation of function with increasing preload rather than afterload [4]. Our study showed ASD patients have higher RVEF than PH patients. In the early stages of disease, RV adaptation is hypertrophy, which is beneficial for RV systolic function. However, this adaptive response to loading conditions will change the RV shape and myofiber architecture, and subsequently, cardiac contractility will decrease. A previous study suggested this remodeling of RV corresponding to various loading conditions is significantly related to RV function and mechanics [12]. Therefore, developing a suitable modality for assessing RV morphology and function in different disease cohorts is very important in daily clinical practice.

Echocardiography is the first-line, readily available method for evaluating RV structure and function. Conventional 2D echocardiography is the most widely used method. There are many parameters suitable for RV assessment in adults, such as FAC, peak systolic velocity (s’) and TAPSE. Among these parameters, TAPSE is the most frequently used. It is simple and reproducible to evaluate RV function, and its prognostic value has been verified. A recent research found that TAPSE ≤14 mm indicated a worse outcome in HF patients with reduced LVEF and dilated cardiomyopathy [13]. TAPSE is a preload-dependent parameter, and our study demonstrated that TAPSE significantly increased in ASD patients due to the volume-overloaded condition. However, TAPSE only reflects the longitudinal orientation motion of the RV free wall, and a few sources of measurement bias must be considered. Speckle tracking has been widely studied in the LV in the past two decades, and it has recently been introduced to assess RV function [14]. Strain derived from this echocardiographic technique has been proposed as a relevant parameter for risk stratification of patients with PH. Studies have shown that RV global longitudinal strain is a potential early marker for prognosis [8, 14]. However, longitudinal strain may suffer from preload dependency. This study demonstrated that G-RVLS and FW-RVLS significantly increased in ASD patients compared to PH patients and controls. This single orientation of RV contraction may not define coupling with the pulmonary circulation. Due to the complex anatomy and physiology of the RV, accurate measurements of the RV remain a challenge.

CMRI is known as the gold standard for measuring RV volume and function. However, its low accessibility and high cost prevent it from being a routine diagnostic technique. With recent technique advances, 3DE can evaluate RV morphology and function without geometric assumptions. Therefore, it provides an accurate method to quantify RV volumes and EF with high reproducibility and correlation with CMRI. In this study, RV three-dimensional data originated from a new technique by using a knowledge-based reconstruction (KBR) database, which had already shown excellent accuracy and reproducibility in calculating RV volumes according to CMRI [11]. In our study, ASD patients with chronic volume overload showed significantly larger RV volumes both at end-diastole and end-systole and stroke volume than PH patients and controls, even after adjusting for BSA. Similarly, we found that RVEF increased significantly in ASD patients. According to the Frank-Starling law, RV contractile function increases as the preload increases in a reasonable volume, which is an effective compensatory mechanism for altered hemodynamic status. A previous study in hemodialysis patients also showed that RV 3D volumetric and functional parameters are affected by acute preload changes [15]. However, in our study, RVEF derived from 3D in PH patients with pressure overload was significantly lower than that in ASD patients and controls. The RV is particularly afterload sensitive, so the RVEF is decreased because of higher PVR in PH patients. A study including corrected Fallot anomaly or pulmonary stenosis patients performed by Trzebiatowska-Krzynska *et al*. [16] demonstrated that 3DE successfully identified all patients with RV dilatation according to CMRI. In addition, the reproducibility analysis results showed that limits of agreement of 3D data, such as EDV, ESV and RVEF, were narrow in this research population. Our study also demonstrated good reproducibility of this new 3D technique and was suitable for differentiating morphology and functional changes in RV responses to different overload conditions.

Under physiological conditions, the pulmonary vascular bed maintains relatively lower resistance and matches the RV contractility, which maintains favorable RV-PA coupling. However, in PH patients, this balance is disrupted, and RV-PA uncoupling occurs. In recent years, several RV-PA coupling parameters obtained noninvasively by echocardiography have been validated [6, 7, 13]. Since these parameters simultaneously include both the status of RV systolic function and the pulmonary vascular loading conditions, it will improve our understanding of the effects of different overload conditions on the RV in patients.

TAPSE/PASP was proposed as a comprehensive parameter for assessing right heart contractile performance and cardiopulmonary functional status. Tello *et al*. [17] conducted a study including severe idiopathic and thromboembolic PH patients and showed that the TAPSE/PASP ratio was able to predict RV-PA uncoupling with a sensitivity of 87.5% and specificity of 75.9%, at a cut-off value of TAPSE/PAS *p* < 0.31 mm/mmHg. In our study, PH patients had the lowest TAPSE/PASP ratio among the three groups (0.49 ± 0.20 vs 0.62 ± 0.17 and 0.86 ± 0.18, *p* = 0.0025 and *p* < 0.0001, respectively). This result is mainly ascribed to the higher pulmonary vascular resistance and more significant impairment of RV function in PH patients. Previous research demonstrated that TAPSE/SPAP ratio had the strongest relationship with RV functional status after cardiac resynchronization therapy (CRT) [18]. Saeed S *et al*. [19] concluded that a TAPSE/PASP index <0.49 mm/mmHg is strongly associated with all-cause mortality in patients with moderate or severe tricuspid regurgitation. However, Schmeisser *et al*. [20] found that compared with TAPSE/PASP, TAPSE is a more available and valid surrogate parameter for RV functional change in HFrEF patients. In our study, the TAPSE/PASP ratio only showed a moderately negative correlation with 3D parameters in the PH group. These different conclusions may result from the different demographics and co-morbidities in the various populations.

The other parameter for noninvasively assessing RV-PA coupling is RVLS/PASP, which has been proven to have prognostic value in HFrEF patients. A recent study found that low values of G-RVLS/PASP and FW-RVLS/PASP are independently associated with a higher risk of cardiovascular events and can predict nonresponse to CRT [21]. In our cohort, G-RVLS/PASP and FW-RVLS/PASP were significantly lower and showed medium-high correlations with 3D data in PH patients. Though the RV longitudinal strain is relatively loading independent, it reflects just the longitudinal motion of RV, and cannot completely determine RV function.

The SV/ESV ratio, as a volumetric method of RV-PA coupling, had been investigated in PH patients, and showed a good correlation with the reference measurements of arterial and ventricular elastance obtained with RHC and CMRI [22]. Similar results were found in our study, in which the SV/ESV ratio had a strong correlation with RVEF in both ASD and PH patients (r = 0.8703, *p* < 0.001 and r = 0.9388, *p* < 0.001, respectively). Previous research has indicated SV/ESV is also an independent predictor of outcome in RV over pressure-loading conditions [9, 23]. In our study, the results demonstrated that the PH group had a lower SV/ESV ratio than the ASD group and controls. We speculate that the result is mainly due to long-term afterload causing RV function impairment, which is more significant in PH patients. Therefore, RV-PA uncoupling may occur more frequently in pressure-loading conditions. The SV/ESV ratio allows us to understand the cardiopulmonary vascular unit as a whole system and is more sensitive to RV dysfunction.

## 5. Study Limitations

Several limitations of this study should be noted. First, the RV complex geometry presents challenge to assess RV function by echocardiography. Global RV function is composed by different directional motion, and the relative importance of these components should be investigated [12]. In this study, TAPSE or RV strain refers only to the longitudinal orientation motion of more complex RV contraction, which may sometimes misguide clinicians. However, in our study, RV morphology and function were evaluated by 3D echocardiography, which is considered the most accurate technique. Our results also showed that most RV-PA coupling parameters have good or moderate correlations with 3D data. Second, this research was derived from a single center with a relatively small sample size, meaning the results must be confirmed in a larger prospective study.

## 6. Conclusions

Non-invasive RV-PA coupling parameters derived from echocardiography appear similar, but not identical, in profiles involving different loading conditions. These parameters, such as TAPSE/PASP, G-RVLS/PASP and FW-RVLS/PASP, decrease not only in pressure-overloaded but also volume-overloaded patients. The volume method of SV/ESV shows a strong correlation with RV function, and G-RVLS/PASP, FW-RVLS/PASP share a similar degree of correlation. The TAPSE/PASP has just a moderate correlation with RV 3D volumetric and functional indices.

## Data Availability

The datasets used and/or analysed during the current study are available from the corresponding author on reasonable request.

## Abbreviations

ASD, atrial septal defect; 3DE, three-dimensional echocardiography; FAC, fractional area change; FW-RVLS, right ventricular free wall longitudinal strain; G-RVLS, right ventricular global longitudinal strain; CMRI, cardiac magnetic resonance imaging; PASP, pulmonary artery systolic pressure; PH, pulmonary hypertension; RV, right ventricular; RV-PA, right ventricle-pulmonary arterial; RHC, right heart catheterization; TAPSE, tricuspid annular plane systolic excursion.
